# Designing optimized industrial process analysers for closed loop control

**DOI:** 10.1155/S1463924691000263

**Published:** 1991

**Authors:** Bernard Grevesmuehl, Cynthia Kradjel, Hanno Kellner

**Affiliations:** Bran + Luebbe GmbH 4 Werkstrasse Postfach 13 60 Norderstedt D 2000 Germany

## Abstract

Manufacturers are now looking closely at ways of optimizing ‘quality’ and increasing process efficiency while reducing manufacturing costs. Near infra-red (NIR) technology is a popular solution to this challenge: it provides manufacturers with rapid and reliable in-process analysis and thousands of systems have already been installed in the food, chemical, pharmaceutical and agricultural markets.

For over 10 years, NIR has been successfully applied to at-line process analysis. Rugged and easy-to-operate filter analysers are traditionally located in the control room–process operators can then ‘grab samples’ and obtain results in less than a minute. There are many practical advantages to using at-line filter systems. Products from many lines can be run on one system, and, since there is no direct process interface, installation, operation and maintenance are quite simple.

Many manufacturers, however, are now striving to achieve on-line closed loop control, in these cases the benefit of obtaining continuous measurement is well worth the effort required to automate the analysis.


Journal of Automatic Chemistry, Vol. 13, No. 4 (July–August 1991), pp. 139–142

### Designing optimized industrial process analysers for closed loop control

**Bernard Grevesmuehl, Cynthia Kradjel and Hanno Kellner**
*Bran + Luebbe GmbH, 4 Werkstrasse, Postfach 13 60, D 2000 Norderstedt, Germany*

*Manufacturers are now looking closely at ways of optimizing 'quality' and increasing process efficiency while reducing manufacturing costs. Near infra-red (NIR) technology is a popular solution to this challenge: it provides manufacturers with rapid and reliable in-process analysis and thousands of systems have already been installed in the food, chemical, pharmaceutical and agricultural markets.*

*For over 10 years, NIR has been successfully applied to at-line process analysis. Rugged and easy-to-operate filter analysers are traditionally located in the control room—process operators can then 'grab samples' and obtain results in less than a minute. There are many practical advantages to using at-line filter systems. Products from many lines can be run on one system, and, since there is no direct process interface, installation, operation and maintenance are quite simple.*

*Many manufacturers, however, are now striving to achieve on-line closed loop control, in these cases the benefit of obtaining continuous measurement is well worth the effort required to automate the analysis.*

The installation of analytical equipment into existing production facilities can be broken down into several phases. *Project definition* determines which parameters will be used to monitor the performance of the factory; these parameters must be correctable when they run out of range by specific actions in the process machinery.

*Analyser design* must be optimized for factory conditions: for example, food manufacturers must ensure that foreign material, such as glass or bacteria, cannot enter the process stream, and chemical manufacturers often face great variability in environmental conditions and must protect against explosions.

The *Sample interface* is designed to take into account factory conditions and the optical appearance of the sample. Sample types vary greatly from fine or coarse powders to viscous, dark, clear or corrosive liquids; each sample type will exhibit different optical characteristics that range from transparent to slightly scattering to highly scattering. The analyser can be configured with reflectance, transmission or transflectance for these differing sample types.

The following examples are actual installations of liquid and solid applications, using the Bran + Luebbe InfraAlyzer 600. In some cases, the system is used in the non-contact mode and in other examples it is used in the contact mode.

The optical unit is based on patented Reference-Sample-Dark optics (RSD). This optical configuration is called 'precision scanning' because, for each wavelength measured, several real-time readings of the gold-plated integrating sphere and the dark current are also measured. The design enables constant check against changes in the environment or optics of the system (see figure 1).

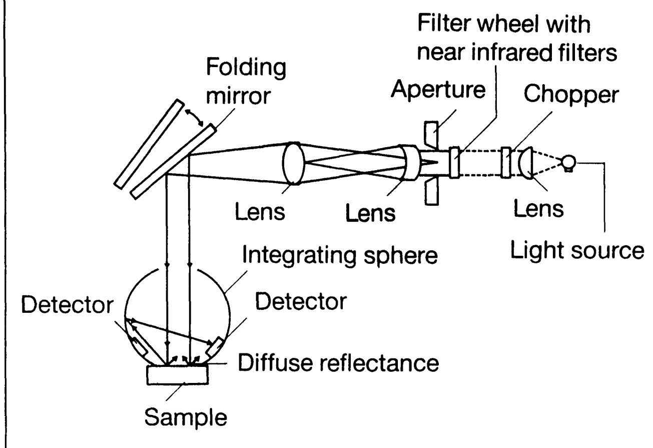

*Figure 1. The InfraAlyzer 600 patented 'RSD' optical configuration.*

The wavelengths are achieved by high-precision band-pass filters which are mounted on a filter wheel. The optical head is a separate from the electronic unit. The optics module can be connected either directly to a liquid cell or sampling device or mounted above the production line. The electronic unit fits into a standard 19 in rack.

### Liquid applications

The first Bran + Luebbe on-line process liquid analysis systems were installed in the mid 1980s.

The InfraAlyzer 600 is successfully used in the sugar processing industry; calibrations have been developed in co-operation with the University of Ferrara, Italy for the determination of Brix value and degree of polarization in process liquids, including raw, thin and thick juices. These parameters are used to optimize efficiency when processing the beet to crystalline sugar. The Brix value is related to the total solids content and the degree of polarization is related to the ratio of sugars, such as saccharose, glucose and fructose. The juice is pumped through a stainless-steel pipe to a suitable point in the

0142–0453/91 $3.00 © 1991 Taylor & Francis Ltd.

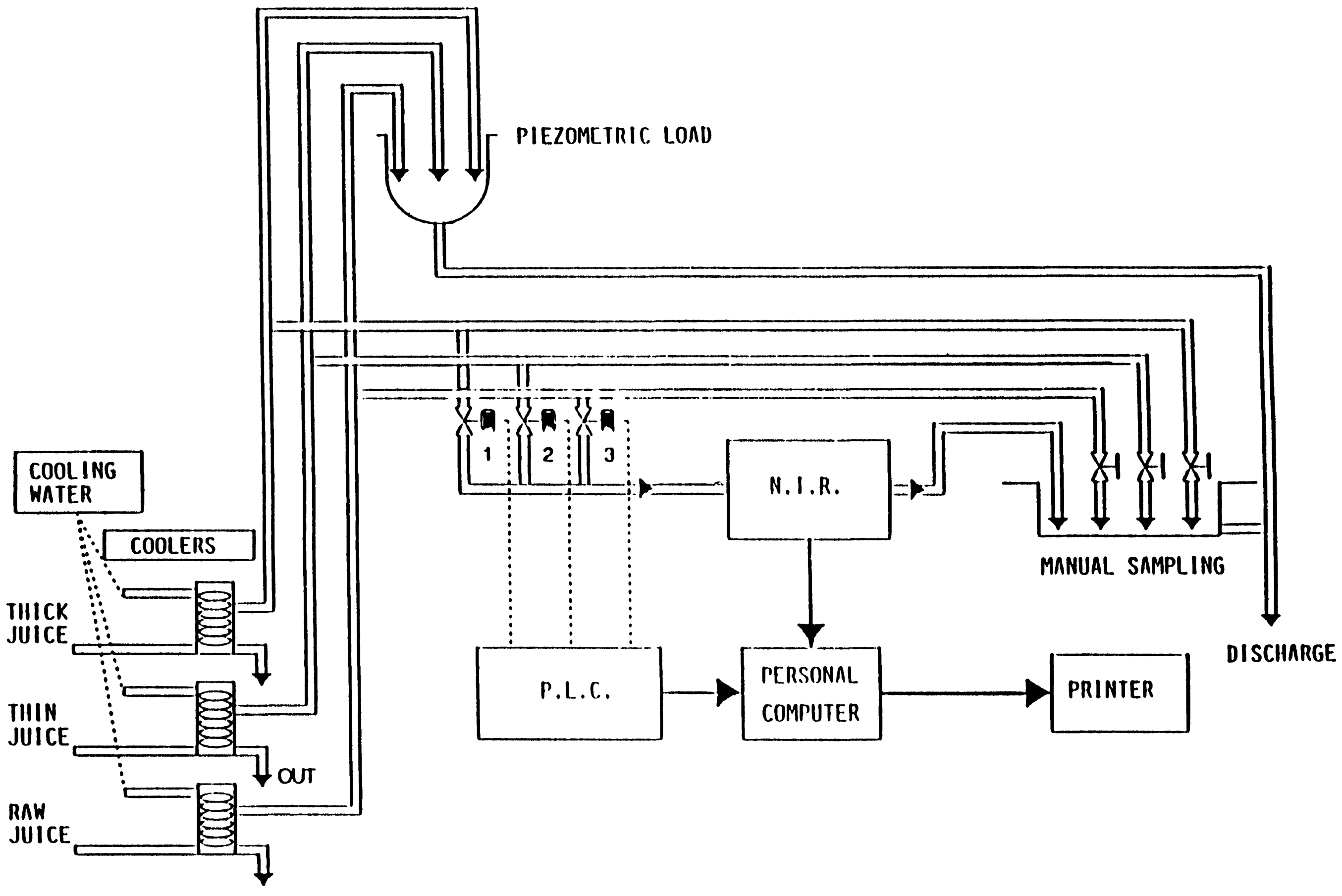

*Figure 2. Analysis scheme for BRIX and degree of polarization in sugar.*

plant and then thermostatted at 50 °C. The juice flows continuously through a small tank to ensure a homogeneous stream and a constant load. Pneumatic valves, which are controlled by a PLC, are used to direct the liquid to the thermostatted liquid manifold for NIR analysis and then to discharge. The process includes an automatic rinsing stage (see figure 2). Thus sugar manufacturers can easily blend process streams to achieve the 'target value' every time.

Another on-line process liquid measurement system that operates in a closed loop control process is being used in brewing. The system controls a blending station with a PLC system, water being continuously added to a high-gravity beer line. In this case, the InfraAlyzer 600 is equipped with a special liquid cell. This process line is kept under high pressure to eliminate carbon dioxide degassing, so the liquid cell is designed to withstand pressures up to 6 bar absolute. The on-line unit is connected to a bypass line where the beer is pumped at 150 ml/min through the liquid cell. The analyser is interfaced with a precision dosing pump and a temperature control system for the sample loop. The normal temperature range of the sample is 0–3 °C but the system is suitable for samples in the range 0–15 °C and containing about 1–3 volumes of carbon dioxide. The original gravity is analysed at the same level of accuracy as the laboratory NIR analysers. The reproducible and controlled sample presentation allows for this high degree of accuracy. The standard error is about ±0·25% in the calibration range of 26–100° Saach. Alcohol is simultaneously determined with a standard error of about 0·15% in the concentration range of 0–6·6% v/v. A minimum of 60 sample analyses can be carried out per hour (see figure 3).

### Solid applications

Solid sampling systems can be either contact or non-contact; each has its advantages and disadvantages. In general, contact analysers require more complicated sample interfacing and are not easily adaptable to pasteous products. However, they are ideally suited for fine powders. They can often achieve very high levels of precision and accuracy because the sample is presented in a very reproducible fashion and process variables are controlled. Non-contact systems are typically mounted above a product. This is practical for sticky or adhesive products. However, the system will be susceptible to variations in the distance of the sample from the analyser.

The earliest contact system for powders was developed in conjunction with the Federated Milling and Baking Research Association (FMBRA) in the UK. Flour is diverted through a slip-stream pipe and automatically pressed against the window. The sample is stationary while analysis is taking place, and then sent back to the line by a compressed air line. The flour is measured for moisture and protein. When necessary, the system signals a valve to open and automatically add gluten to the flour to increase the protein content.

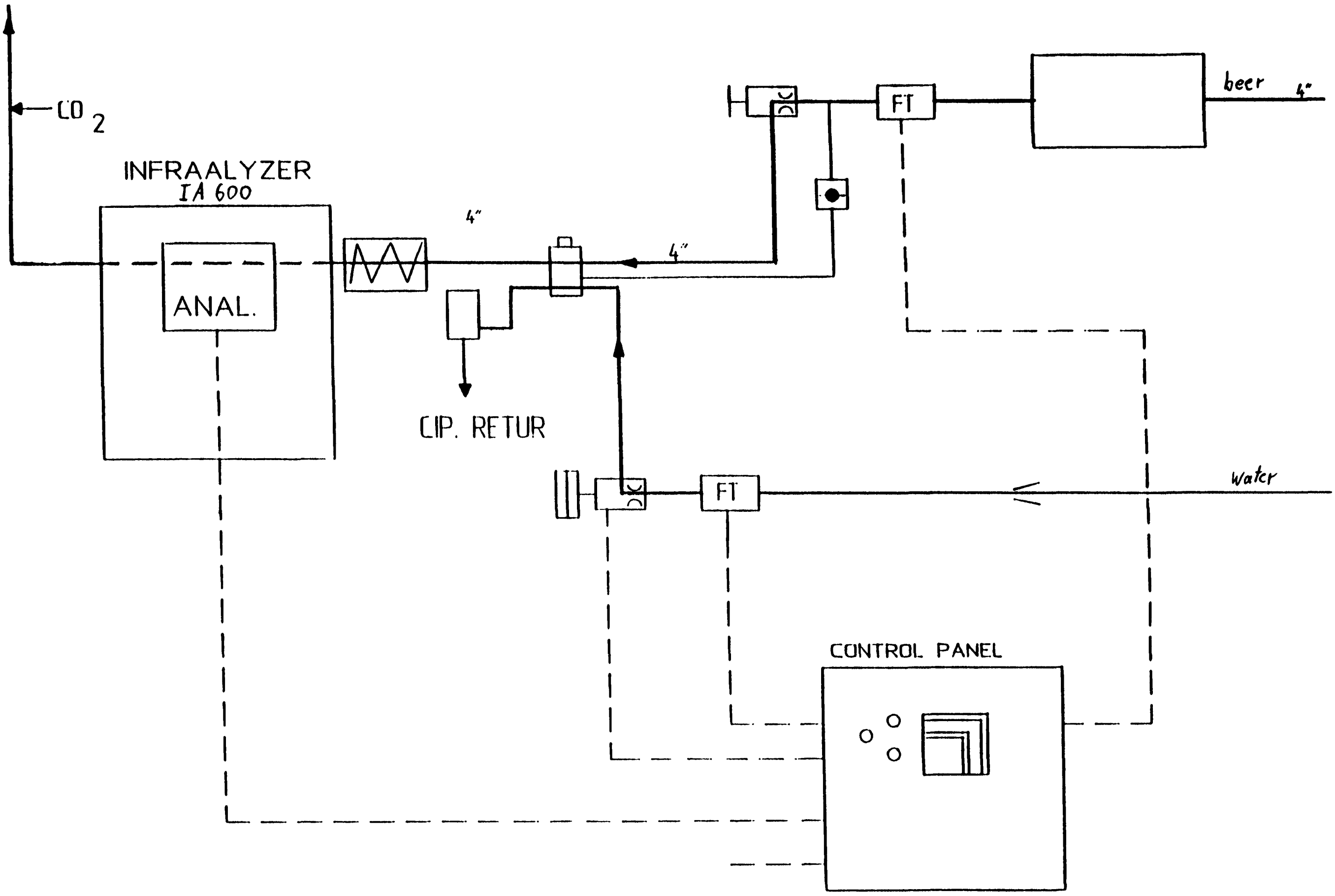

*Figure 3. Configuration for continuous original gravity in beer.*

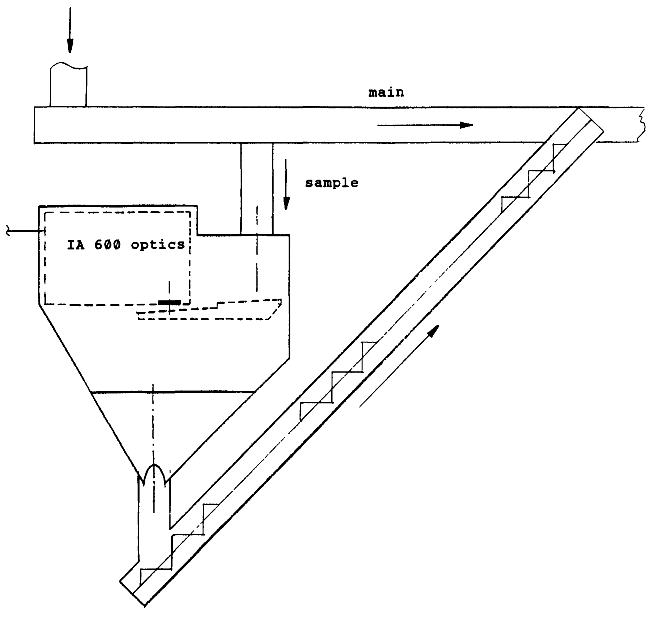

*Figure 4. Fat and moisture determination in milk-powder.*

Fat, moisture and protein are critical parameters in milk-powder processing. Since milk-powder can be adhesive, Bran + Luebbe has developed a non-contact sampling approach for this application.

The optical module is fitted into a compact sampling system and mounted on a bypass pipe after the main milk-powder fluid bed. The powder travels to a point underneath the optics module via a magnetic vibrator. A uniform density and constant from the optical window is achieved through precise setting of the magnetic vibrator. The flow stops during the measurement cycle. When analysis is completed, the sample is returned to the main line through a spiral conveyor. Fat and protein are determined to an accuracy of ±0·1% and moisture is measured to an accuracy of ±0·02%. Approximately 60 samples are analysed per hour. The sample temperature ranges between 18–22 °C (see figure 4).

The last example illustrates the use of the InfraAlyzer 600 for the analysis of fat in cream cheese. The optics module is mounted vertically, reading cream cheese at 80 °C as it comes out of a pressure stabilized bypass and runs down a spade-like dispenser. The optical module is sealed against dust and water. The electronic unit is located in a control room and is connected to an IBM-PC for Statistical Process Control. The fat content ranges from 46–53% with an accuracy of ±0·25%.

There are many other applications of the InfraAlyzer 600 for on-line NIR Process Analysis, including moisture in textiles, additives to thermoplastics, and fat and protein in meat. For applications that require full spectral scanning, Bran + Luebbe has introduced two High Performance Crystal Spectroscopy systems: the Infra-PRIME (Process Integrated Monitoring Equipment), based on the technology of Acousto-Optical Tunable

Scanning (AOTS); and the InfraPROVER, a polarizing interferometer optimized for the NIR region of the spectrum.

### Note

The applications described here are systems that were co-developed with users. Pending the final conclusions of these installations, Bran + Luebbe will announce in September 1991 which complete systems will be sold as Bran + Luebbe standard products. Currently, Bran + Luebbe offers the InfraAlyzer 600 as a basic unit and sampling interfaces are developed in conjunction with the individual user.

